# Changes in behaviors after diagnosis of type 2 diabetes and 10-year incidence of cardiovascular disease and mortality

**DOI:** 10.1186/s12933-019-0902-5

**Published:** 2019-08-01

**Authors:** Jean Strelitz, Amy L. Ahern, Gráinne H. Long, Clare E. Boothby, Nicholas J. Wareham, Simon J. Griffin

**Affiliations:** 1 MRC Epidemiology Unit, Institute of Metabolic Science, Cambridge Biomedical Campus, University of Cambridge School of Clinical Medicine, Box 285, Cambridge, CB2 0QQ UK; 2 AstraZeneca Pharmaceuticals, Cambridge, UK; 3 Primary Care Unit, Institute of Public Health, University of Cambridge School of Clinical Medicine, Cambridge, UK

**Keywords:** Type 2 diabetes, Cardiovascular disease, Lifestyle, Diet, Physical activity, Alcohol use

## Abstract

**Background:**

Large changes in health behaviors achieved through intensive lifestyle intervention programs improve cardiovascular disease (CVD) risk factors among adults with type 2 diabetes. However, such interventions are not widely available, and there is limited evidence as to whether changes in behaviors affect risk of CVD events.

**Methods:**

Among 852 adults with screen-detected type 2 diabetes in the *ADDITION*-*Cambridge* study, we assessed changes in diet, physical activity, and alcohol use in the year following diabetes diagnosis. Participants were recruited from 49 general practices in Eastern England from 2002 to 2006, and were followed through 2014 for incidence of CVD events (n = 116) and all-cause mortality (n = 127). We used Cox proportional hazards regression to estimate hazard ratios (HR) for the associations of changes in behaviors with CVD and all-cause mortality. We estimated associations with CVD risk factors using linear regression. We considered changes in individual behaviors and overall number of healthy changes. Models adjusted for demographic factors, bodyweight, smoking, baseline value of the health behavior, and cardio-protective medication use.

**Results:**

Decreasing alcohol intake by ≥ 2 units/week was associated with lower hazard of CVD vs maintenance [HR: 0.56, 95% CI 0.36, 0.87]. Decreasing daily calorie intake by ≥ 300 kcal was associated with lower hazard of all-cause mortality vs maintenance [HR: 0.56, 95% CI 0.34, 0.92]. Achieving ≥ 2 healthy behavior changes was associated with lower hazard of CVD vs no healthy changes [HR: 0.39, 95% CI 0.18, 0.82].

**Conclusions:**

In the year following diabetes diagnosis, small reductions in alcohol use were associated with lower hazard of CVD and small reductions in calorie intake were associated with lower hazard of all-cause mortality in a population-based sample. Where insufficient resources exist for specialist-led interventions, achievement of moderate behavior change targets is possible outside of treatment programs and may reduce long-term risk of CVD complications.

*Trial registration* This trial is registered as ISRCTN86769081. Retrospectively registered 15 December 2006

**Electronic supplementary material:**

The online version of this article (10.1186/s12933-019-0902-5) contains supplementary material, which is available to authorized users.

## Background

Behavioral and lifestyle factors are key contributors to cardiovascular complications among adults with diabetes [1]. Despite the fact that changes in diet, physical activity, smoking and alcohol use are recognized as a cornerstone of type 2 diabetes treatment [2], there is limited evidence as to whether changes in these behaviors affect risk of cardiovascular disease (CVD) events. Behavioral intervention trials have demonstrated short-term improvements in cardiovascular risk factors among adults with diabetes randomized to specialist-led physical activity and diet interventions [3–7]. For example, in the *Action for Health in Diabetes* (*Look AHEAD*) trial, > 2 metabolic equivalent (MET) increases in physical fitness were associated with improvements in HbA_1c_, high-density lipoprotein (HDL) and triglycerides [8], although there were no apparent associations with CVD incidence [9]. However, the majority of type 2 diabetes patients do not receive behavioral treatment. Results from selective trial cohorts may not be generalizable to broader patient populations, and behavior changes achieved in such trials may not be realistic in the absence of a costly specialist-led intervention.

There is limited evidence from population-based cohorts as to the CVD benefits of behavior change. In an observational study of adults with self-reported diabetes enrolled in the *Nurses’ Health Study* and *Health Professionals Follow*-*up Study*, participants who made at least 2 healthy behavior changes after diabetes diagnosis had lower 10-year hazard of CVD compared to participants who made no changes. However, this study did not assess the impact of individual behavior changes on CVD and did not determine behavior change targets that may be effective to reduce CVD risk [10]. In the *ADDITION*-*Cambridge* study, we showed that participants who increased physical activity or decreased alcohol consumption in the year following diabetes diagnosis had lower 5-year hazard of CVD, and the total number of healthy behavior changes also had a protective association with CVD [11]. Despite the suggestion that improvements in behaviors decrease risk of CVD, this study had a low number of CVD events due to the relatively short follow up, which precluded our ability to consider magnitude of behavior change, instead focusing on whether participants increased or decreased their behaviors. Participants in these two studies did not receive an intensive behavioral intervention and results suggest that modest and achievable behavior changes could reduce risk of CVD events in people with type 2 diabetes. However, further research is needed to establish meaningful and clinically relevant behavior change targets.

We have expanded on the prior analyses in the *ADDITION*-*Cambridge* study [12] by considering magnitudes of behavior changes and by including a further 5 years of follow-up for CVD and mortality outcomes. We have also examined the association between behavior changes and CVD risk factors [HbA_1c_; systolic and diastolic blood pressure; triglycerides; total- and LDL-cholesterol]. Our objective was to assess the associations of behavior changes in the year following diabetes diagnosis with CVD risk factors and 10-year incidence of CVD events and all-cause mortality, in a cohort of adults with screen-detected type 2 diabetes who had not received intensive behavioral treatment.

## Methods

### Study population

*ADDITION*-*Cambridge* (ISRCTN86769081) is a population-based study of screening for type 2 diabetes, followed by a cluster-randomized trial comparing a multifactorial treatment approach with routine care. The present study is an observational analysis of the *ADDITION*-*Cambridge* trial cohort. Study participants were adults 40–69 years old recruited from 49 general practices (GPs) in eastern England [12]. Eligible participants were adults at high risk of diabetes, as identified from electronic records using a validated risk score [13]. Of 33,539 eligible high-risk individuals, 74% attended stepwise screening from 2002 to 2006 [14], where type 2 diabetes was diagnosed using the 1999 World Health Organization criteria [15]. All 867 adults identified to have type 2 diabetes consented to participate in the study. Practices were randomized to either multifactorial treatment (n = 26), which included more frequent consultations and supplemental educational materials [12], or to follow current UK national guidelines for diabetes management [16–18] (n = 23). There was no behavioral component of the intervention. One hundred and twenty-one participants who were concurrently enrolled in the *ADDITION*-*Plus* sub-study also received 6 facilitator-led meetings to promote change in key behaviors [19], however this intervention did not result in changes in behaviors or risk factors [20]. Informed consent was obtained from all individual participants included in the study. Ethical approval was obtained from local research ethics committees (Cambridge, ref:01/063; Huntingdonshire, ref:00/609; Peterborough and Fenland, ref:P01/95; West Essex, ref:1511-0103; North and Mid Essex, ref:MH395 MREC02/5/54; West Suffolk, ref:03/002; Hertfordshire and Bedfordshire, ref:EC03623; and the Eastern Multi-Centre Research Ethics Committee, ref:02/5/54).

### Measurements

Anthropometric measures, blood lipids, and HbA_1c_ were assessed, and questionnaires were administered, at the time of diabetes diagnosis (baseline) and 1 year later [12]. Physical activity was defined as past year total physical activity energy expenditure (net MET hours per day) and was assessed using the validated European Prospective Investigation into Cancer (EPIC)-Norfolk Physical Activity Questionnaire [21]. Diet was assessed with a validated food frequency questionnaire [22]. Plasma vitamin C, an objective marker of fruit and vegetable consumption, was measured using fluorometric assay [12]. Alcohol intake was ascertained via self-report. Socio-demographic information (age, sex, occupation, age left full-time education, and ethnicity), smoking, and prescribed medication use were self-reported. Occupational socioeconomic status (SES) was categorized according to the Registrar General’s classification system: ‘professional, managerial and technical’, ‘skilled-manual and non-manual’, and ‘partly skilled or unskilled’ [23].

### CVD and mortality outcomes

Incidence of CVD and all-cause mortality was ascertained from the date of diabetes diagnosis until December 31st, 2014. The composite CVD outcome included the following endpoints: cardiovascular mortality, myocardial infarction, stroke, non-traumatic amputation, invasive cardiovascular revascularization procedures, and peripheral revascularization procedures. Possible CVD outcomes were identified via searches of GP notes, hospital records, electrocardiograms, laboratory results, death certificates, autopsies, and the Myocardial Infarction National Audit Project (MINAP) [24]. Participants were followed for mortality surveillance by the Office of National Statistics using National Health Service patient numbers. All outcome events were independently adjudicated.

### Statistical analyses

Of the 867 adults enrolled in the *ADDITION*-*Cambridge* study, we were unable to include 15 participants who had a CVD event or died during the year following diabetes diagnosis, as this is when the behavior changes were assessed. Therefore, this study included 852 participants with 116 CVD events and 127 all-cause mortalities. Numbers of participants included in individual analyses varied due to missing information on health behaviors and other covariates included in the models.

#### Behavior change and CVD incidence and mortality

The behavior variables were categorized into groups representing increase, decrease or maintenance of each behavior. Cut points were determined based on the distributions of change in each behavior from baseline to 1 year in order to compare changes which could be reasonably translated into patient recommendations that would be achievable in the target population. The categorizations were as follows: average daily physical activity (increase or decrease by ≥ 2 MET hours/day vs. < 2 MET hours/day change); daily total energy intake (increase or decrease by ≥ 300 kcal/day vs. < 300 kcal/day change); daily intake of fat as a percentage of total energy (increase or decrease by ≥ 4% vs. < 4% change); daily fiber intake (increase or decrease by ≥ 3 g/day vs. < 3 g/day change); plasma vitamin C (increase or decrease by ≥ 10 µmol/l vs. < 10 µmol/l change); and alcohol consumption (increase or decrease by ≥ 2 units/week vs. < 2 units/week change). We were unable to assess associations with change in cigarette smoking because few participants changed their smoking status during the study period.

A health behavior change score summarized the number of healthy behavior changes in the year following diabetes diagnosis [11]: one point was assigned for increasing physical activity; decreasing or abstaining from alcohol consumption; increasing both daily fiber and vitamin C intake; and decreasing both daily energy and total proportion of fat intake. Higher behavior change scores reflect adoption of more healthy behaviors. We performed a sensitivity analysis with an alternate behavior change score, which awarded 0.5 points for improvement in each of the dietary factors instead of awarding one point for improving both total energy and fat, or both fiber and vitamin C. Analyses of behavior change scores were restricted to the 597 study participants with complete information on all relevant health behaviors at baseline and 1 year. To address potential bias due to informative missing information, we assessed whether any measured covariates predicted missingness of the behavior change variables and performed a sensitivity analysis accounting for missing behavior change information using multiple imputation, as described below.

We used Cox proportional hazards regression to estimate hazard ratios for 10-year CVD incidence and for all-cause mortality, by individual behavior changes and by behavior change score. The time scale was time since diabetes diagnosis. Participants were at risk for an incident event beginning 1 year after diabetes diagnosis until first incident CVD event, death, or the end of the study period on 31 December, 2014. We assessed adherence to the proportional hazards assumption by modelling an interaction term between the natural log of time in study and each covariate, which indicated no departures from proportional hazards. We identified confounders a priori using a directed acyclic graph [25]: age at baseline (continuous), sex (female, male), trial group (intensive treatment, routine care), baseline occupational SES (‘professional, managerial and technical’, ‘skilled-manual and non-manual’, and ‘partly skilled or unskilled’), age left full-time education (< 16 years, 16–18 years, > 18 years), baseline body mass index (BMI) (kg/m^2^) (coded as a continuous variable with a quadratic term), smoking reported at 1 year (current, former, never), and use of antihypertensive (yes, no), glucose-lowering (yes, no) and lipid-lowering medication (yes, no) reported at 1 year. Models for the individual behavior changes were also adjusted for the baseline value of the behavior. Clustering of participants within GPs was accounted for using a robust cluster variance estimator [26].

We conducted sensitivity analyses in which we (i) used multiple imputation to assess robustness of our results to missing information on health behaviors, (ii) adjusted for change in weight in the year following diabetes diagnosis since weight loss during this period was associated with lower hazard of CVD events [27], and (iii) mutually adjusted for all behavior changes in the models for each individual behavior change. The multiple imputation models included covariates for behavior change score, sex, SES, education, baseline BMI, smoking, treatment group, anti-hypertensive, glucose-lowering, and lipid-lowering medication use at 1 year, outcome status, and the Nelson–Aalen estimate of cumulative hazard. Hazard ratios were estimated from 20 imputed datasets.

#### Behavior changes and CVD risk factors

We used linear regression to assess the relationship between behavior changes in the year following diabetes diagnosis and CVD risk factors [HbA_1c_; systolic and diastolic BP; triglycerides; total- and LDL-cholesterol] measured 1 year after diabetes diagnosis. Models were adjusted for age at baseline, sex, SES, education, trial group, baseline BMI, smoking at 1 year, and relevant cardio-protective medication use at 1 year [i.e. glucose-lowering medication for HbA_1c_, anti-hypertensive medication for BP, and lipid-lowering medication for lipid levels]. Models for individual behavior changes were also adjusted for the baseline value of the behavior. Clustering within GPs was accounted for as in the proportional hazards models described above.

## Results

Among 852 study participants, mean age at diagnosis (SD) was 61.0 (7.2) years, 61% of participants were male, 97% were white, 33% were in a managerial or professional occupation, and 51% left full-time education after age 16. Participants were followed for an average of 9.7 years from the date of diabetes diagnosis. Characteristics and measured CVD risk factors were generally similar among men and women (Table 1), so analyses were conducted among the full cohort. Use of lipid- and glucose-lowering medication increased across the study period. However, prevalence of glucose-lowering medication use was generally low at 1 year (29% in women and 32% in men), likely due to the fact that participants were in the early stages of diabetes progression and mean HbA_1c_ at baseline was quite low (Table 1). Of the 116 incident CVD events during the study period, 39 were revascularizations, 29 were CVD deaths, 29 were strokes, 18 were MI, and 1 was a non-traumatic amputation.

**Table 1:** Characteristics of type 2 diabetes patients at time of diagnosis (baseline) and 1 year later

	Female (n = 330)		Male (n = 522)	
	Baseline	1 year	Baseline	1 year
Cohort characteristics				
BMI (kg/m^2^), mean (SD)	34.7 (6.0)	33.1 (6.1)	32.7 (5.4)	31.7 (5.2)
Missing, n (%)	2 (0.6%)	50 (15.2%)	3 (0.6%)	74 (14.2%)
Weight (kg), mean (SD)	88.2 (16.1)	84.3 (16.5)	98.5 (17.8)	95.6 (17.1)
N missing	1 (0.3%)	50 (15.2%)	3 (0.6%)	74 (14.2%)
Smoking, n (%)				
Current	47 (14.3%)	33 (11.5%)	104 (19.9%)	77 (17.0%)
Former	123 (37.4%)	113 (39.4%)	268 (51.3%)	243 (53.8%)
Never	159 (48.3%)	141 (49.1%)	150 (28.7%)	132 (29.2%)
Missing, n (%)	1 (0.3%)	43 (13.0%)	0 (0.0%)	70 (13.4%)
CVD risk factors				
HbA_1c_ (%), mean (SD)	7.2 (1.6)	6.5 (0.8)	7.4 (1.7)	6.5 (0.9)
mmol/mol, mean	55.2	47.5	57.4	47.5
Missing, n (%)	12 (3.6%)	55 (17.0%)	9 (1.7%)	76 (14.6%)
Blood pressure (mmHg), mean (SD)				
Systolic	140 (20)	133 (18)	143 (20)	138 (18)
Diastolic	80 (9)	77 (9)	83 (11)	80 (10)
Missing, n (%)	2 (0.6%)	52 (15.8%)	0 (0.0%)	73 (14.0%)
Lipids (mmol/l), mean (SD)				
Total cholesterol	5.6 (1.1)	4.7 (0.9)	5.2 (1.1)	4.4 (1.0)
Missing, n (%)	10 (3.0%)	51 (15.5%)	9 (1.7%)	73 (14.0%)
LDL	3.4 (1.0)	2.6 (0.8)	3.2 (1.0)	2.5 (0.9)
Missing, n (%)	15 (4.5%)	58 (17.6%)	35 (6.7%)	90 (17.2%)
Triglyceride	1.9 (1.1)	1.8 (1.0)	2.2 (1.8)	2.0 (1.5)
Missing, n (%)	10 (3.0%)	51 (15.5%)	10 (1.9%)	73 (14.0%)
Medication use, n (%)				
Glucose-lowering				
Yes	1 (0.3%)	81 (28.7%)	3 (0.6%)	143 (32.1%)
No	327 (99.7%)	201 (71.3%)	519 (99.4%)	303 (67.9%)
Missing, n (%)	2 (0.6%)	48 (14.5%)	0 (0.0%)	76 (14.6%)
Antihypertensive				
Yes	212 (64.6%)	207 (73.4%)	278 (53.3%)	297 (66.6%)
No	116 (35.4%)	75 (26.6%)	244 (46.7%)	149 (33.4%)
Missing, n (%)	2 (0.6%)	48 (14.5%)	0 (0.0%)	76 (14.6%)
Lipid-lowering				
Yes	70 (21.3%)	186 (66.0%)	136 (26.1%)	292 (65.5%)
No	258 (78.7%)	96 (34.0%)	386 (73.9%)	154 (34.5%)
Missing, n (%)	2 (0.6%)	48 (14.5%)	0 (0.0%)	76 (14.6%)
Health behaviors				
Total physical activity (MET hours/day), median (Q1, Q3)	8.9 (5.6, 12.0)	8.7 (6.1, 12.4)	10.6 (6.9, 16.3)	11.4 (7.3, 17.4)
Missing, n (%)	2 (0.6%)	42 (12.7%)	1 (0.2%)	69 (13.2%)
Energy intake (kcal/day), mean (SD)	1832 (650)	1660 (645)	2057 (734)	1762 (570)
Missing, n (%)	5 (1.5%)	43 (13.0%)	7 (1.3%)	72 (13.8%)
Fiber intake (g/day), mean (SD)	17.6 (6.9)	19.8 (11.1)	16.4 (6.6)	18.3 (11.1)
Missing, n (%)	5 (1.5%)	43 (13.0%)	7 (1.3%)	72 (13.8%)
Fat as percentage of energy intake, mean (SD)	32.7 (6.0)	30.4 (6.0)	33.2 (6.3)	31.3 (6.3)
Missing, n (%)	5 (1.5%)	43 (13.0%)	7 (1.3%)	72 (13.8%)
Plasma vitamin C (µmol/l), mean (SD)	56.7 (23.4)	60.9 (24.9)	49.7 (21.7)	50.4 (22.5)
Missing, n (%)	41 (12.4%)	62 (18.8%)	45 (8.6%)	86 (16.5%)
Alcohol (mean units/week), mean (SD)	3.3 (5.9)	3.0 (5.1)	10.3 (13.2)	9.0 (11.4)
Missing, n (%)	8 (2.4%)	48 (14.5%)	6 (1.1%)	74 (14.2%)

*ADDITION*-*Cambridge* (N = 852) 2002–2014

Among participants with non-missing covariate information, 218 (31%) increased their physical activity by ≥ 2 MET hours/day, 361 (53%) decreased their alcohol intake by ≥ 2 units/week, 303 (44%) decreased their total energy intake by ≥ 300 kcal/day, 256 (37%) decreased their daily fat intake by ≥ 4%, 250 (37%) increased their fiber intake by ≥ 3 g/day, and 199 (34%) increased their plasma vitamin C levels ≥ 10 µmol/l between baseline and 1 year (Table 2). After summing the number of healthy changes in the behavior change score, 36 (6%) made no healthy changes between baseline and 1 year, 145 (26%) made 1 healthy change, 217 (38%) made 2 healthy changes, and 167 (30%) made 3 or 4 healthy changes (Table 2). Those who increased total physical activity, fiber intake, and plasma vitamin C during the first year in study had lower baseline values of these behaviors compared to participants who decreased these behaviors. Similarly, participants who decreased daily total energy, fat intake, and alcohol use during the first year in study had higher baseline values for these measures (Table 2).

**Table 2:** Hazard ratios for the association of behavior changes from baseline to 1 year and CVD and all-cause mortality at 10 years follow-up

Behavior change	Baseline, mean (SD)	1 year, mean (SD)	Mean change (SD)	N cases/N total^a^	HR [95% CI]^b^ CVD	N cases/N total^a^	HR [95% CI]^b^ all-cause mortality
Total physical activity (MET hours/day)							
Increased ≥ 2 MET hours	10.1 (6.6)	16.4 (8.8)	6.3 (4.5)	30/218	1.10 [0.61, 1.98]	27/218	0.87 [0.47, 1.60]
Maintained within 2 MET hours	9.1 (5.3)	9.1 (5.4)	0.0 (1.1)	36/277	1	41/277	1
Decreased ≥ 2 MET hours	16.6 (8.8)	10.1 (6.3)	− 6.5 (5.5)	26/198	0.92 [0.55, 1.55]	25/198	0.90 [0.50, 1.63]
Alcohol (mean units/week)							
Decreased ≥ 2 units or abstained	8.5 (13.6)	5.1 (9.3)	− 3.4 (6.1)	38/361	0.56 [0.36, 0.87]	52/362	1.06 [0.67, 1.67]
Maintained within 2 units	5.5 (6.4)	5.5 (6.3)	0.0 (0.8)	38/213	1	25/214	1
Increased ≥ 2 units	9.2 (8.2)	15.6 (13.4)	6.4 (7.7)	15/101	0.72 [0.34, 1.53]	15/101	1.13 [0.67, 1.93]
Energy intake (kcal/day)							
Decreased ≥ 300 kcal	2376 (721)	1590 (490)	− 786 (472)	38/303	0.78 [0.46, 1.33]	34/303	0.56 [0.34, 0.92]
Maintained within 300 kcal	1682 (469)	1653 (482)	− 29 (164)	37/279	1	44/279	1
Increased ≥ 300 kcal	1617 (546)	2284 (829)	668 (301)	16/102	1.36 [0.69, 2.69]	14/102	1.19 [0.56, 2.52]
Fat as percentage of energy intake (%)							
Decreased ≥ 4%	35.6 (5.4)	27.4 (5.7)	− 8.3 (3.9)	37/256	1.03 [0.64, 1.65]	38/256	0.95 [0.59, 1.53]
Maintained within 4%	32.4 (5.4)	32.0 (5.2)	− 0.3 (2.3)	40/314	1	37/314	1
Increased ≥ 4%	28.0 (6.2)	35.7 (5.7)	7.8 (3.5)	14/114	0.87 [0.41, 1.85]	17/114	1.22 [0.58, 2.57]
Fibre intake (g/day)							
Increased > 3 g/day	15.0 (5.5)	24.1 (15.7)	9.0 (14.1)	32/250	0.94 [0.57, 1.55]	32/250	0.99 [0.57, 1.71]
Maintained within 3 g/day	16.0 (5.5)	16.1 (5.5)	0.1 (1.7)	41/296	1	44/296	1
Decreased ≥ 3 g/day	22.8 (7.9)	15.5 (5.7)	− 7.4 (5.0)	18/138	1.36 [0.63, 2.94]	16/138	0.93 [0.42, 2.06]
Plasma vitamin C (µmol/l)							
Increased > 10 µmol/l	41.6 (18.2)	67.4 (23.8)	25.8 (16.3)	22/199	0.68 [0.41, 1.13]	29/199	1.18 [0.69, 2.01]
Maintained within 10 µmol/l	53.1 (21.6)	53.0 (22.0)	0.0 (5.5)	34/236	1	31/236	1
Decreased ≥ 10 µmol/l	65.5 (21.4)	40.6 (19.0)	− 24.9 (12.5)	21/156	0.97 [0.58, 1.65]	17/156	0.85 [0.41, 1.77]
Behavior change score							
0 changes	n/a	n/a	n/a	9/36	1	5/37	1
1 change	n/a	n/a	n/a	25/145	0.60 [0.26, 1.37]	16/146	0.68 [0.23, 2.00]
2 changes	n/a	n/a	n/a	23/217	0.39 [0.18, 0.82]	40/220	1.15 [0.45, 2.89]
3–4 changes	n/a	n/a	n/a	19/167	0.42 [0.19, 0.95]	14/170	0.46 [0.13, 1.54]

ADDITION-Cambridge 2002–2014 (N = 852)

^a^The total number of participants with nonmissing information on all covariates in the full model

^b^Models are adjusted for age, sex, SES, education, BMI at baseline, smoking at 1 year, baseline value of the behavior, treatment group, and use of antihypertensive, glucose-lowering or lipid-lowering medications at 1 year

Decreasing or abstaining from alcohol consumption in the year following diabetes diagnosis was associated with lower hazard of CVD at 10 years [HR (95% CI) decrease ≥ 2 units vs maintaining alcohol intake: 0.56 (0.36, 0.87)]. Decreasing total energy intake by ≥ 300 kcal per day was associated with lower hazard of all-cause mortality vs maintaining intake [HR (95% CI) 0.56 (0.34, 0.92)] (Table 2). Total number of healthy behavior changes was associated with a lower CVD hazard at 10 years [HR (95% CI) 2 changes vs 0 changes: 0.39 (0.18, 0.82); 3–4 changes vs. 0 changes: 0.42 (0.19, 0.95)].

Participants who made at least 3 healthy behavior changes had lower cholesterol and LDL at 1 year of follow-up compared to participants who made no healthy changes. Those who decreased daily energy intake by ≥ 300 kcal had lower LDL compared to those who maintained energy intake, and those who decreased fat intake had lower diastolic blood pressure compared to those who maintained their intake. Participants who increased plasma vitamin C had lower triglycerides compared to those who maintained plasma vitamin C levels (Table 3).

**Table 3:** Beta coefficients and 95% confidence intervals from multivariable linear regression models of the association of behavior changes in the year following diabetes diagnosis and cardiovascular risk factors measured 1 year after diagnosis

	HbA_1c_ (%)		Cholesterol (mmol/l)		LDL (mmol/l)		Triglycerides (mmol/l)		Systolic BP (mmHg)		Diastolic BP (mmHg)	
	N^a^	β^b^ [95% CI]	N^a^	β^b^ [95% CI]	N^a^	β^b^ [95% CI]	N^a^	β^b^ [95% CI]	N^a^	β^b^ [95% CI]	N^a^	β^b^ [95% CI]
Physical activity												
Increased ≥ 2 MET hours/day	211	− 0.05 [− 0.16, 0.05]	212	-0.02 [− 0.18, 0.14]	206	− 0.03 [− 0.16, 0.10]	212	− 0.02 [− 0.14, 0.10]	213	− 1.41 [− 4.40, 1.57]	213	0.08 [− 1.76, 1.93]
Maintained within 2 MET hours/day	266	1	271	1	263	1	271	1	271	1	271	1
Decreased ≥ 2 MET hours/day	189	0.05 [− 0.08, 0.19]	190	-0.00 [− 0.18, 0.17]	183	− 0.03 [− 0.21, 0.14]	190	0.04 [− 0.07, 0.15]	191	2.34 [− 1.36, 6.05]	191	1.06 [− 0.28, 2.40]
Alcohol												
Decreased ≥ 2 units/week or abstained	350	0.10 [− 0.02, 0.21]	352	− 0.05 [− 0.18, 0.08]	343	− 0.02 [− 0.14, 0.10]	352	− 0.01 [− 0.11, 0.09]	354	− 1.38 [− 4.50, 1.73]	354	− 1.32 [− 3.25, 0.61]
Maintained within 2 units/week	206	1	210	1	203	1	210	1	210	1	210	1
Increased ≥ 2 units/week	97	− 0.11 [− 0.33, 0.11]	97	0.16 [− 0.04, 0.36]	94	0.05 [− 0.11, 0.21]	97	0.02 [− 0.10, 0.14]	97	2.61 [− 1.75, 6.97]	97	0.96 [− 1.40, 3.32]
Energy intake												
Decreased ≥ 300 kcal	295	− 0.14 [− 0.32, 0.03]	297	− 0.08 [− 0.22, 0.06]	290	− 0.13 [− 0.23, − 0.03]	297	− 0.03 [− 0.14, 0.07]	296	1.95 [− 2.13, 6.03]	296	0.16 [− 1.63, 1.95]
Maintained within 300 kcal	267	1	271	1	262	1	271	1	273	1	273	
Increased > 300 kcal	95	0.03 [− 0.15, 0.22]	96	0.06 [− 0.14, 0.25]	91	− 0.06 [− 0.21, 0.08]	96	0.05 [− 0.10, 0.20]	97	3.72 [− 0.32, 7.76]	97	0.94 [− 1.46, 3.33]
Fat as % of energy intake												
Decreased ≥ 4%	249	− 0.10 [− 0.25, 0.05]	250	− 0.08 [− 0.23, 0.06]	245	− 0.05 [− 0.18, 0.07]	250	− 0.05 [− 0.14, 0.05]	252	0.43 [− 2.94, 3.79]	252	− 1.78 [− 3.52, − 0.03]
Maintained within 4%	298	1	304	1	290	1	304	1	304	1	304	1
Increased ≥ 4%	110	0.04 [− 0.14, 0.22]	110	0.13 [− 0.08, 0.35]	108	0.17 [− 0.01, 0.35]	110	− 0.02 [− 0.16, 0.11]	110	2.61 [− 1.25, 6.47]	110	0.65 [− 2.04, 3.34]
Fibre intake												
Increased > 3 g/day	239	0.07 [− 0.08, 0.22]	239	− 0.05 [− 0.19, 0.09]	231	− 0.04 [− 0.18, 0.09]	239	0.02 [− 0.09, 0.12]	241	0.17 [− 2.66, 2.99]	241	0.09 [− 1.62, 1.80]
Maintained within 3 g/day	285	1	290	1	281	1	290	1	291	1	291	1
Decreased ≥ 3 g/day	133	0.12 [− 0.09, 0.34]	135	0.06 [− 0.09, 0.22]	131	− 0.08 [− 0.22, 0.06]	135	0.10 [− 0.01, 0.20]	134	3.09 [− 0.43, 6.62]	134	1.79 [− 0.37, 3.94]
Plasma vitamin C												
Increased > 10 µmol/l	199	− 0.08 [− 0.25, 0.10]	199	− 0.06 [− 0.31, 0.19]	194	− 0.00 [− 0.21, 0.21]	199	− 0.16 [− 0.28, − 0.05]	199	− 2.65 [− 6.32, 1.02]	199	− 1.34 [− 3.23, 0.55]
Maintained within 10 µmol/l	231	1	236	1	227	1	236	1	236	1	236	1
Decreased ≥ 10 µmol/l	154	0.08 [− 0.12, 0.29]	156	− 0.03 [− 0.23, 0.18]	152	− 0.03 [− 0.23, 0.16]	156	− 0.02 [− 0.11, 0.08]	155	− 0.67 [− 4.15, 2.81]	155	− 0.87 [− 3.05, 1.31]
Behavior change score												
0 changes	35	1	36	1	34	1	36	1	36	1	36	1
1 change	144	− 0.23 [− 0.69, 0.23]	145	− 0.16 [− 0.44, 0.12]	139	− 0.11 [− 0.36, 0.15]	145	− 0.01 [− 0.20, 0.18]	144	− 0.78 [− 6.42, 4.86]	144	− 1.08 [− 4.33, 2.17]
2 changes	215	− 0.20 [− 0.58, 0.18]	217	− 0.40 [− 0.64, − 0.15]	211	− 0.18 [− 0.38, 0.02]	217	− 0.13 [− 0.32, 0.06]	217	1.07 [− 4.23, 6.38]	217	− 2.47 [− 5.61, 0.67]
3–4 changes	165	− 0.11 [− 0.49, 0.27]	167	− 0.40 [− 0.64, − 0.15]	165	− 0.21 [− 0.42, − 0.01]	167	− 0.14 [− 0.33, 0.05]	167	− 2.05 [− 7.59, 3.49]	167	− 2.63 [− 5.86, 0.61]

ADDITION-Cambridge 2002–2014

^a^The total number of participants with nonmissing information on all covariates in the full model

^b^Models are adjusted for age, sex, BMI at baseline, smoking at 1 year, SES, education, treatment group, relevant cardio-protective medication use, and baseline value of the behavior

Missing behavior change information was associated with sex, SES and higher baseline BMI; unskilled workers were more likely to have missing information compared to professional workers, and women were more likely to have missing information compared to men. Accounting for missing information using multiple imputation did not meaningfully change the observed associations between behavior changes and CVD or mortality (Additional file 1). Results were also robust to adjusting for weight change from baseline to 1 year (Additional file 2) and adjusting for individual behavior changes (Additional file 3). The associations between behavior change score and CVD were similar when considering an alternate behavior change scoring method where each dietary change counted for 0.5 points (Additional file 4).

## Discussion

In this population-based study of adults with screen-detected diabetes, modest changes in health behaviors in the year following diabetes diagnosis were associated with a 44–58% lower hazard of CVD 10 years after diabetes diagnosis. Specifically, reducing alcohol intake by ≥ 2 units/week was associated with an estimated 44% lower hazard compared to maintaining alcohol intake. Those who reduced total energy intake by ≥ 300 kcal/day also had 44% lower hazard of all-cause mortality compared to those who maintained their intake, but this was not associated with CVD events. Those who made at least 2 overall healthy changes had lower hazard of CVD compared to those who made no healthy changes. The observed associations between behaviors, CVD and mortality were independent of weight change and baseline behaviors, and were robust to sensitivity analyses.

Our study is the first to have assessed the impacts of moderate changes in health behaviors relative to maintenance of behaviors after diabetes diagnosis to show that moderate changes that were achievable with no behavioral intervention may reduce incidence of CVD events. The study highlights the important role of lifestyle management in diabetes treatment, which is particularly relevant in light of results from the Diabetes Remission Clinical Trial (DiRECT) which demonstrated the benefits of lifestyle change on diabetes status [28] and the Action to Control Cardiovascular Risk in Diabetes (ACCORD) trial which showed that intensification of glucose-lowering treatment may increase mortality [29]. The results of this study are supported by results from the *Nurses’ Health Study* and the *Health Professionals Follow*-*up Study*, in which improvements in a behavior score (reflecting changes in diet, physical activity and alcohol consumption) from before to after diabetes diagnosis was associated with a 21% lower hazard of CVD events at 10 years [10]. A previous analysis in *ADDITION*-*Cambridge* which considered changes in behaviors after diabetes diagnosis and 5-year incidence of CVD showed similar associations between alcohol reduction and ≥ 2 behavior changes and incidence of CVD [11].

In our study, reduction in alcohol consumption was associated with lower hazard of CVD events. Few studies have assessed changes in alcohol consumption and CVD incidence, although one study showed that short-term abstention from alcohol was associated with improvement in insulin resistance and CVD risk factors [30]. The mechanisms by which alcohol consumption impacts CVD risk remains unclear. Metabolism of ethanol from alcoholic beverages may lead to generation of reactive oxygen species in the blood, which can contribute to atherogenesis [31] and may thereby increase risk of a future CVD event. However, several studies have reported protective associations between light to moderate alcohol consumption, CVD and mortality [32–36], although these studies did not assess changes in alcohol consumption. In contrast, a Mendelian randomization meta-analysis showed a lower risk of CVD associated with lower alcohol consumption [37]. Our result is in agreement with a previous study in *ADDITION*-*Cambridge* which showed that participants who reduced or abstained from alcohol intake had an estimated 62% lower 5-year hazard of CVD compared to participants who increased their alcohol intake [11]. Our sensitivity analyses suggested that the protective association between alcohol reduction and CVD was independent of weight loss. We also did not observe any clear associations between change in alcohol intake and CVD risk factors. Therefore, weight loss and improvement in traditional CVD risk factors may not be the primary mechanism by which alcohol reduction may lead to lower incidence of CVD.

In the current study, increases in total physical activity of ≥ 2 MET hours per day were not associated with risk of CVD. This is in contrast to studies that have found protective associations of increases in physical activity and CVD [9, 11]. Differences in results may be related to the degree of physical activity achieved, maintenance of physical activity levels over time, and differences in error in the tools used to measure physical activity. The *Look AHEAD* trial showed a non-statistically significant 32% lower 10-year hazard of CVD among adults with diabetes who had > 2 MET increases in physical fitness in 1 year [9]. However, in the *Look AHEAD* trial, changes in fitness levels were achieved via intensive lifestyle intervention, and study participants may have been more able to engage in physical activity compared to the general diabetes patient population [38]. We speculate that the amount and duration of change in physical activity achieved among the *ADDITION* cohort may not have been sufficient to yield reduction in CVD events. There is substantial research supporting a biological role of physical activity to improve cardiovascular health. Past studies have shown that exercise improves endothelial function [39], improves cardiovascular risk factors including glycaemia and lipid levels [40] and reduces incidence of CVD [41]. We did not objectively measure physical activity or physical fitness but relied on self-reported activity. Misreport of physical activity likely contributed to misclassification of changes in physical activity, which would bias our results toward the null and reduce our ability to detect any association between physical activity and CVD.

There are several limitations to consider when interpreting results from this study. There were baseline differences in health behaviors by category of behavior change, which may have affected our ability to detect associations between changes in behaviors and the outcomes of interest; however, we adjusted for baseline values of the behaviors to address this confounding. We considered relative increases or decreases vs maintenance of behaviors, but could not assess finer categorizations of behavior changes due to limitations of sample size. This study used validated questionnaires to assess diet, physical activity and alcohol intake [21, 22], and objective measurement of plasma vitamin C, however self-reported exposure information may have been misclassified due to recall error or other misreport. The EPIC Physical Activity Questionnaire has shown moderate within-individual repeatability (correlation coefficients > 0.60) [21], and the amount of error in the tool may impede our ability to discern small changes in physical activity over time. Alcohol intake is often underreported [42, 43], and total energy intake may be underreported by individuals with higher BMI [44]. However, because we have assessed within-individual changes in the behaviors rather than between-individual changes, our results may be less sensitive to imprecision in the absolute measure of the behavior. Misclassification of behavior changes would likely bias results towards the null, as we do not anticipate that misclassification would be related to the outcomes.

Possible health differences between participants who made behavior changes versus those who did not may have introduced unmeasured confounding. There were no apparent differences in demographic and lifestyle characteristics at baseline after stratifying by number of healthy behavior changes, though there were small differences in distributions of SES (Additional file 5). However, we adjusted for confounding by SES in all analyses. There were also no differences in CVD risk factors at baseline (Additional file 5), which supports the interpretation that the observed associations between behavior change score and CVD were not due to differences in underlying cardiovascular risk between these groups. While our results suggest that behavior changes in the first year after diabetes diagnosis are potentially important for CVD reduction independent of weight loss, the degree of maintenance of change may also be important; we were unable to consider modification by maintenance of behavior changes at 5 years in study as the number of CVD events between 5 and 10 years was small. Analyses of behavior changes and CVD events were subject to censoring due to the competing risk of non-CVD death, and we addressed this by censoring participants at the date of CVD event, death, or the end of the study period, whichever came first.

The *ADDITION* cohort is a population-based sample, and all individuals determined to be eligible during screening enrolled in the study. This affords generalizability to the target population of adults in Eastern England with a new diagnosis of type 2 diabetes. Due to the screening-based nature of this study, we were able to capture behavior changes during the period immediately following diabetes diagnosis and assessed whether achievable changes during this period may be beneficial to reduce long-term disease burdens. However, because participants were screen-detected to have diabetes, many participants were in the early stages of diabetes progression and mean HbA_1c_ among the cohort was quite low (7%). This study had repeat measurement of behaviors and is one of few studies to have assessed long-term associations of behavior change after diabetes diagnosis with CVD and mortality [10, 11]. We identified modest behavior changes that were associated with estimated lower hazards of CVD, which may be useful to inform interventions for behavior changes among newly diagnosed patients where resources cannot support behavioral treatment. We achieved 99.8% CVD and mortality ascertainment and all events were independently adjudicated. Furthermore, sensitivity analyses showed that our results were robust to missing information on health behaviors, and that the observed associations were independent of weight loss.

A substantial number of participants spontaneously made moderate changes to their behavior following diabetes diagnosis. However, receiving a diagnosis of diabetes may not be sufficient to trigger behavior changes in most patients and so behavioral interventions at the point of diagnosis could support more people to make changes [45]. Intervening early in diabetes progression to control risk factors may help to avoid complications associated with intensification of diabetes treatment and may reduce long-term CVD events [46]. The study results suggest that making small changes across a few behaviors may reduce CVD risk; these changes may be translated to a decrease of 2 units of alcohol per week (e.g. one glass of wine), a decrease in daily calorie intake by 300 kcal (e.g. one muffin), and an increase in 2 MET hours per day of physical activity (e.g. 30 min of casual walking or cycling).

## Conclusion

This is the first study to identify achievable targets for behavior changes to potentially reduce CVD risk among adults with diabetes, in the absence of an intensive behavioral intervention. Participants who made at least two healthy behavior changes in the year following diabetes diagnosis had 58–61% lower hazard of CVD events at 10 years compared to participants who made no healthy changes. Reducing alcohol consumption and decreasing calorie intake in the year following diabetes diagnosis were respectively associated with lower hazard of CVD and all-cause mortality at 10 years. Where specialist-led interventions are unavailable, clinicians and policy-makers may consider emphasizing moderate behavior change targets following diabetes diagnosis, as changes during this period may yield long-term reductions in CVD events.

## Additional files

**Additional file 1.** Hazard ratios for the associations of health behavior changes from baseline to 1 year and 10-year incidence of CVD and mortality, with multiple imputation* to account for missing data (N = 852).

**Additional file 2.** Hazard ratios for the association of health behavior changes from baseline to 1 year and 10-year CVD and mortality incidence, adjusting for weight change from baseline to 1 year (N = 725).

**Additional file 3.** Hazard ratios for the associations of health behavior changes from baseline to 1 year and 10-year CVD and mortality incidence, adjusting for individual behavior changes (N = 565*).

**Additional file 4.** Hazard ratios for the associations of a behaviour change scoring method giving equal weight to dietary changes, and CVD and all-cause mortality. ADDITION-Cambridge 2002–2014 (N = 565*).

**Additional file 5.** Baseline characteristics of participants by number of overall healthy behavior changes in the year following diabetes diagnosis. ADDITION-Cambridge 2002–2014.

## Data Availability

The datasets used and/or analyzed during the current study are available from the corresponding author on reasonable request.

## Abbreviations

ACCORD: Action to Control Cardiovascular Risk in Diabetes
ADDITION: Anglo–Danish–Dutch Study of Intensive Treatment in People with Screen-Detected Diabetes in Primary Care
BMI: body mass index
BP: blood pressure
CVD: cardiovascular disease
DiRECT: Diabetes Remission Clinical Trial
EPIC: European Prospective Investigation into Cancer
HbA_1c_: haemoglobin A1c
GP: general practice
LDL: low-density lipoprotein
Look AHEAD: Action for Health in Diabetes
MET: metabolic equivalents
MINAP: Myocardial Infarction National Audit Project
MRC: Medical Research Council
NHS: National Health Service
NIHR: National Institute for Health Research
SES: socioeconomic status
