# Supplementary material for: Changes in behaviors after diagnosis of type 2 diabetes and 10-year incidence of cardiovascular disease and mortality
Source: Cardiovasc Diabetol. 2019 Aug 1;18:98. doi: 10.1186/s12933-019-0902-5 (PMC6670127; doi:10.1186/s12933-019-0902-5)
Supplement: Supplementary file 1 — Additional file 1. Hazard ratios for the associations of health behavior changes from baseline to 1 year and 10-year incidence of CVD and mortality, with multiple imputation* to account for missing data (N = 852). [file 12933_2019_902_MOESM1_ESM.docx]

| Additional File 1. Hazard ratios for the associations of health behavior changes from baseline to 1 year and 10-year incidence of CVD and mortality, with multiple imputation* to account for missing data (N=852). | | |
| --- | --- | --- |
| Behavior change score‡ | HR [95%CI] CVD events† | HR [95%CI] All-cause mortality† |
| 0 changes | 1 | 1 |
| 1 change | 0. 48 [0.25, 0.93] | 0.65 [0.24, 1.75] |
| 2 changes | 0.36 [0.18, 0.73] | 1.08 [0.42, 2.77] |
| 3-4 changes | 0.38 [0.18, 0.80] | 0.42 [0.15, 1.16] |
| *The multiple imputation model included covariates for behavior change score, sex, SES, education, baseline BMI, smoking, treatment group, anti-hypertensive, glucose-lowering, and lipid-lowering medication use at 1 year, outcome status, and the Nelson-Aalen estimate of cumulative hazard | | |
| †Models are adjusted for age, sex, SES, BMI at baseline, smoking, treatment group, and use of antihypertensive, glucose-lowering or lipid-lowering medications at 1 year | | |
| ‡Behavior change score awards 1 point each for any improvement between baseline and one year in physical activity or alcohol intake, and 1 points for improvement in both total energy and proportion fat intake, and 1 point for improvement in both fiber intake and plasma vitamin C levels. | | |
