# Supplementary material for: Changes in behaviors after diagnosis of type 2 diabetes and 10-year incidence of cardiovascular disease and mortality
Source: Cardiovasc Diabetol. 2019 Aug 1;18:98. doi: 10.1186/s12933-019-0902-5 (PMC6670127; doi:10.1186/s12933-019-0902-5)
Supplement: Supplementary file 2 — Additional file 2. Hazard ratios for the association of health behavior changes from baseline to 1 year and 10-year CVD and mortality incidence, adjusting for weight change from baseline to 1 year (N = 725). [file 12933_2019_902_MOESM2_ESM.docx]

| Additional File 2. Hazard ratios for the association of health behavior changes from baseline to 1 year and 10-year CVD and mortality incidence, adjusting for weight change from baseline to 1 year (N=725). | | |  |
| --- | --- | --- | --- |
| Behavior change | HR [95%CI] CVD events | HR [95%CI] All-cause mortality | |
| Total physical activity (MET hrs/day) | | | |
| Increased ≥2 MET hrs | 1.08 [0.57, 2.03] | 0.86 [0.47, 1.56] | |
| Maintained within 2 MET hrs | 1 | 1 | |
| Decreased ≥2 MET hrs | 0.94 [0.56, 1.58] | 0.85 [0.47, 1.54] | |
| Alcohol (Mean units/week) |  |  | |
| Decreased ≥2 units or abstained | 0.58 [0.38, 0.89] | 1.12 [0.71, 1.78] | |
| Maintained within 2 units | 1 | 1 | |
| Increased ≥2 units | 0.70 [0.33, 1.47] | 1.03 [0.61, 1.75] | |
| Energy intake (kcal/day) |  |  | |
| Decreased ≥300 kcal | 0.78 [0.45, 1.35] | 0.58 [0.34, 0.98] | |
| Maintained within 300 kcal | 1 | 1 | |
| Increased >300 kcal | 1.34 [0.68, 2.66] | 1.10 [0.50, 2.44] | |
| Fat as percentage of energy intake (%) |  |  | |
| Decreased ≥4% | 1.05 [0.64, 1.72] | 0.93 [0.56, 1.54] | |
| Maintained within 4% | 1 | 1 | |
| Increased ≥4% | 0.92 [0.43, 1.96] | 1.19 [0.56, 2.52] | |
| Fibre intake (g/day) |  |  | |
| Increased >3g/day | 0.94 [0.56, 1.56] | 1.00 [0.57, 1.73] | |
| Maintained within 3g/day | 1 | 1 | |
| Decreased ≥3g/day | 1.42 [0.67, 3.03] | 1.00 [0.46, 2.18] | |
| Plasma Vitamin C (µmol/l) |  |  | |
| Increased >10 µmol/l | 0.69 [0.42, 1.15] | 1.24 [0.74, 2.08] | |
| Maintained within 10 µmol/l | 1 | 1 | |
| Decreased ≥10 µmol/l | 0.99 [0.58, 1.68] | 0.84 [0.40, 1.76] | |
| Behaviour change score |  |  | |
| 0 changes | 1 | 1 | |
| 1 change | 0.62 [0.27, 1.39] | 0.70 [0.24, 2.05] | |
| 2 changes | 0.38 [0.17, 0.82] | 1.23 [0.49, 3.08] | |
| 3-4 changes | 0.44 [0.20, 0.95] | 0.50 [0.15, 1.64] | |
| *The total number of participants with nonmissing information on all covariates in the full model | | |  |
| †Models are adjusted for age, sex, SES, education, BMI at baseline, smoking, weight change between baseline and 1 year, treatment group, baseline value of the health behavior, and use of antihypertensive, glucose-lowering or lipid-lowering medications at 1 year | | |  |
