# Supplementary material for: Changes in behaviors after diagnosis of type 2 diabetes and 10-year incidence of cardiovascular disease and mortality
Source: Cardiovasc Diabetol. 2019 Aug 1;18:98. doi: 10.1186/s12933-019-0902-5 (PMC6670127; doi:10.1186/s12933-019-0902-5)
Supplement: Supplementary file 3 — Additional file 3. Hazard ratios for the associations of health behavior changes from baseline to 1 year and 10-year CVD and mortality incidence, adjusting for individual behavior changes (N = 565*). [file 12933_2019_902_MOESM3_ESM.docx]

| Additional File 3. Hazard ratios for the associations of health behavior changes from baseline to 1 year and 10-year CVD and mortality incidence, adjusting for individual behavior changes (N=565*). | | |
| --- | --- | --- |
| Behaviour change | HR [95%CI] CVD events † | HR [95%CI] All-cause mortality † |
| Total physical activity (MET hrs/day) |  |  |
| Increased ≥2 MET hrs | 1.08 [0.58, 2.00] | 0.86 [0.44, 1.68] |
| Maintained within 2 MET hrs | 1 | 1 |
| Decreased ≥2 MET hrs | 1.13 [0.63, 2.02] | 0.96 [0.50, 1.86] |
| Alcohol (Mean units/week) |  |  |
| Decreased ≥2 units or abstained | 0.55 [0.32, 0.97] | 1.04 [0.61, 1.75] |
| Maintained within 2 units | 1 | 1 |
| Increased ≥2 units | 0.94 [0.45, 1.98] | 1.18 [0.65, 2.13] |
| Energy intake (kcal/day) |  |  |
| Decreased ≥300 kcal | 0.91 [0.50, 1.67] | 0.64 [0.34, 1.22] |
| Maintained within 300 kcal | 1 | 1 |
| Increased >300 kcal | 1.53 [0.74, 3.19] | 1.26 [0.58, 2.73] |
| Fat as percentage of energy intake (%) |  |  |
| Decreased ≥4% | 1.27 [0.73, 2.20] | 0.86 [0.46, 1.59] |
| Maintained within 4% | 1 | 1 |
| Increased ≥4% | 0.86 [0.38, 1.95] | 1.10 [0.48, 2.52] |
| Fibre intake (g/day) |  |  |
| Increased >3g/day | 0.77 [0.45, 1.33] | 0.81 [0.41, 1.58] |
| Maintained within 3g/day | 1 | 1 |
| Decreased ≥3g/day | 1.90 [0.77, 4.69] | 1.19 [0.40, 3.50] |
| Plasma Vitamin C (µmol/l) |  |  |
| Increased >10 µmol/l | 0.70 [0.42, 1.16] | 1.26 [0.72, 2.22] |
| Maintained within 10 µmol/l | 1 | 1 |
| Decreased ≥10 µmol/l | 1.00 [0.58, 1.71] | 0.74 [0.36, 1.51] |
| *The total number of participants with nonmissing information on all covariates in the model | | |
| †Models are adjusted for age, sex, SES, education, BMI at baseline, smoking, category of change in each health behavior, baseline value of the behavior, treatment group, and use of antihypertensive, glucose-lowering or lipid-lowering medications at 1 year | | |
