# Supplementary material for: Changes in behaviors after diagnosis of type 2 diabetes and 10-year incidence of cardiovascular disease and mortality
Source: Cardiovasc Diabetol. 2019 Aug 1;18:98. doi: 10.1186/s12933-019-0902-5 (PMC6670127; doi:10.1186/s12933-019-0902-5)
Supplement: Supplementary file 4 — Additional file 4. Hazard ratios for the associations of a behaviour change scoring method giving equal weight to dietary changes, and CVD and all-cause mortality. ADDITION-Cambridge 2002–2014 (N = 565*). [file 12933_2019_902_MOESM4_ESM.docx]

| Additional File 4. Hazard ratios for the associations of a behaviour change scoring method giving equal weight to dietary changes, and CVD and all-cause mortality. ADDITION-Cambridge 2002-2014 (N=565*) | | | | |
| --- | --- | --- | --- | --- |
|  | Cases/Total N | HR [95%CI] CVD events † | Cases/Total N | HR [95%CI] All-cause mortality † |
| Behaviour change score |  |  |  |  |
| 0-1 changes | 12/57 | 1 | 5/57 | 1 |
| 1.5-2 change | 28/184 | 0.56 (0.26, 1.23) | 23/184 | 0.97 (0.36, 2.64) |
| 2.5 changes | 13/121 | 0.45 (0.24, 0.84) | 28/121 | 2.12 (0.77, 5.87) |
| 3-4 changes | 23/180 | 0.46 (0.22, 0.99) | 19/203 | 0.75 (0.23, 2.40) |
| *The total number of participants with nonmissing information on all covariates in the full model | | | | |
| †Models are adjusted for age, sex, SES, education, BMI at baseline, smoking at 1 year, treatment group, and use of antihypertensive, glucose-lowering or lipid-lowering medications at 1 year | | | | |
