# Supplementary material for: Changes in behaviors after diagnosis of type 2 diabetes and 10-year incidence of cardiovascular disease and mortality
Source: Cardiovasc Diabetol. 2019 Aug 1;18:98. doi: 10.1186/s12933-019-0902-5 (PMC6670127; doi:10.1186/s12933-019-0902-5)
Supplement: Supplementary file 5 — Additional file 5. Baseline characteristics of participants by number of overall healthy behavior changes in the year following diabetes diagnosis. ADDITION-Cambridge 2002–2014. [file 12933_2019_902_MOESM5_ESM.docx]

| Additional File 5. Baseline characteristics of diabetes patients by number of overall healthy behavior changes in the year following diabetes diagnosis. ADDITION-Cambridge 2002-2014 | | | | | | | | |
| --- | --- | --- | --- | --- | --- | --- | --- | --- |
|  | Behavior change score | | | | | | | |
| Characteristic | 0 | | 1 | | 2 | | 3-4 | |
| Mean age at diagnosis | 62.4 | | 60.4 | | 61.0 | | 61.2 | |
| BMI (kg/m^2^), mean at baseline | 32.4 | | 33 | | 32.7 | | 33.8 | |
| HbA_1c_ (%), mean at baseline | 7.2 | | 7.1 | | 7.4 | | 7.4 | |
| Blood pressure (mmHg), mean at baseline | | |  |  |  |  |  |  |
| Systolic | 141.7 | | 139 | | 142.6 | | 141.5 | |
| Diastolic | 82.1 | | 81.6 | | 80.9 | | 82.6 | |
| Lipids (mmol/l), mean at baseline | |  |  |  |  |  |  |  |
| Total cholesterol | 5.9 | | 5.4 | | 5.4 | | 5.3 | |
| HDL | 1.3 | | 1.2 | | 1.2 | | 1.2 | |
| LDL | 3.5 | | 3.3 | | 3.3 | | 3.3 | |
| Triglyceride | 2.4 | | 2.1 | | 2.1 | | 2.1 | |
| Sex, n (%) |  |  |  |  |  |  |  |  |
| Female | 16 | (42.1) | 53 | (34.4) | 80 | (35.2) | 69 | (38.8) |
| Male | 22 | (57.9) | 101 | (65.6) | 147 | (64.8) | 109 | (61.2) |
| Smoking, n(%) |  |  |  |  |  |  |  |  |
| Current | 5 | (13.2) | 20 | (13.0) | 46 | (20.3) | 28 | (15.7) |
| Former | 24 | (63.2) | 75 | (48.7) | 101 | (44.5) | 77 | (43.3) |
| Never | 9 | (23.7) | 59 | (38.3) | 80 | (35.2) | 73 | (41.0) |
| Age left full-time education |  |  |  |  |  |  |  |  |
| <16 years | 18 | (50.0) | 66 | (43.1) | 101 | (45.1) | 86 | (49.1) |
| 16-18 years | 15 | (41.7) | 66 | (43.1) | 92 | (41.1) | 72 | (41.1) |
| >18 years | 3 | (8.3) | 21 | (13.7) | 31 | (13.8) | 17 | (9.7) |
| Occupational socioeconomic class | | |  |  |  |  |  |  |
| Managerial and professional | 12 | (32.4) | 51 | (33.3) | 86 | (38.6) | 67 | (38.3) |
| Skilled manual and non-manual | 8 | (21.6) | 38 | (24.8) | 42 | (18.8) | 42 | (24.0) |
| Partly skilled or unskilled | 17 | (45.9) | 64 | (41.8) | 95 | (42.6) | 66 | (37.7) |
